# Genotype–phenotype correlation in recessive DNAJB4 myopathy

**DOI:** 10.21203/rs.3.rs-4915388/v1

**Published:** 2024-10-14

**Authors:** Michio Inoue, Divya Jayaraman, Rocio Bengoechea, Ankan Bhadra, Casie A. Genetti, Abdulrahman A. Aldeeri, Betül Turan, Rafael Adrian Pacheco-Orozco, Almundher Al-Maawali, Nadia Al Hashmi, Ayşe Gül Zamani, Emine Göktaş, Sevgi Pekcan, Hanife Tuğçe Çağlar, Heather True, Alan H. Beggs, Conrad C. Weihl

**Affiliations:** Washington University School of Medicine; The Manton Center for Orphan Disease Research, Boston Children’s Hospital, Harvard Medical School; Washington University School of Medicine; Washington University School of Medicine; The Manton Center for Orphan Disease Research, Boston Children’s Hospital, Harvard Medical School; The Manton Center for Orphan Disease Research, Boston Children’s Hospital, Harvard Medical School; Necmettin Erbakan University; Organización Clinica General del Norte; Sultan Qaboos University Hospital, College of Medicine and Health Sciences, Sultan Qaboos University; National Genetic Center, Royal Hospital, Ministry of Health; Necmettin Erbakan University; Necmettin Erbakan University; Necmettin Erbakan University; Necmettin Erbakan University; Washington University School of Medicine; The Manton Center for Orphan Disease Research, Boston Children’s Hospital, Harvard Medical School; Washington University School of Medicine

**Keywords:** DNAJB4, Protein aggregate myopathy, Chaperonopathy, Respiratory failure, Rigid spine syndrome, Heat shock proteins

## Abstract

Protein aggregate myopathies can result from pathogenic variants in genes encoding protein chaperones. DNAJB4 is a cochaperone belonging to the heat shock protein-40 (HSP40) family and plays a vital role in cellular proteostasis. Recessive loss-of-function variants in *DNAJB4* cause myopathy with early respiratory failure and spinal rigidity, presenting from infancy to adulthood. This study investigated the broader clinical and genetic spectrum of DNAJB4 myopathy. In this study, we performed whole-exome sequencing on seven patients with early respiratory failure of unknown genetic etiology. We identified five distinct pathogenic variants in *DNAJB4* in five unrelated families of diverse ethnic backgrounds: three loss-of-function variants (c.547C > T, p.R183*; c.775C > T, p.R259*; an exon 2 deletion) and two missense variants (c.105G > C, p.K35N; c.181A > G, p.R61G). All patients were homozygous. All affected individuals exhibited early respiratory failure, and patients from three families had rigid spine syndrome with axial weakness in proportion to appendicular weakness. Additional symptoms included dysphagia, ankle contractures, scoliosis, neck stiffness, and cardiac dysfunction. Notably, J-domain missense variants were associated with a more severe phenotype, including an earlier age of onset and a higher mortality rate, suggesting a strong genotype–phenotype correlation. Consistent with a loss of function, the nonsense variants presented decreased stability. In contrast, the missense variants exhibited normal or increased stability but behaved as loss-of-function variants in yeast complementation and TDP-43 disaggregation assays. Our findings suggest that DNAJB4 is an emerging cause of myopathy with rigid spine syndrome of variable age of onset and severity. This diagnosis should be considered in individuals presenting with suggestive symptoms, particularly if they exhibit neck stiffness during infancy or experience respiratory failure in adults without significant limb muscle weakness. Missense variants in the J-domain may predict a more severe phenotype.

## Introduction

Protein aggregate myopathies encompass a diverse array of muscle diseases, unified by the presence of protein inclusions, which often contain sarcomeric proteins, within myofibers [1]. These disorders are linked to variants in diverse genes, particularly those encoding chaperones and cochaperones [1, 2]. DNAJB4, a member of the DNAJ proteins (also called HSP40s or J domain proteins), is integral to the heat shock protein chaperone network and plays a vital role in cellular proteostasis [3]. Recent studies have highlighted the association of *DNAJB4* variants with both recessive and dominant forms of myopathy [4, 5]. Recessively inherited DNAJB4 myopathy presents between the ages of one and 45 years and is characterized by respiratory distress and rigid spine syndrome. Dominantly inherited forms typically begin with decreased grip strength, progressing to involve both distal and proximal muscles. Both forms exhibit distinctive pathological features, including the presence of cytoplasmic inclusions and disruption of myofibrillar architecture, suggesting a profound impact on muscle cell function and structure.

Despite these advancements, many aspects of DNAJB4 myopathy remain poorly understood. Although two additional cases carrying *DNAJB4* variants have been identified through reanalysis of whole exome sequencing data [6, 7], comprehensive studies that elucidate consolidated clinical information from multiple patients are scarce. Consequently, the correlation between genotype and phenotype, as well as the underlying pathophysiology of DNAJB4 myopathy, has yet to be fully elucidated.

This study aims to address these critical gaps by analyzing an international cohort of families, thereby identifying new genetic variants from diverse backgrounds and detailing their clinical and genetic spectrum. Through this approach, we aim not only to demonstrate the pathogenicity of these variants through functional analyses but also to enhance our understanding of the molecular mechanisms driving DNAJB4 myopathy.

## Methods

### Patients

We investigated five families with seven affected patients without a genetic diagnosis. The families were from Saudi Arabia (Families 1 and 2), Turkey (Family 3), Colombia (Family 4), and Oman (Family 5). Family 5 was previously reported in the literature [7].

### Genetic testing

The detailed methods of genetic testing performed for each family are listed in the online supplementary materials. Confirmation of variants and segregation testing was performed by Sanger sequencing, except for in Family 4. For Families 2, 3, and 5, variants were identified via reanalysis of whole-exome sequencing data.

### Histological Analyses

Histological analysis was performed by the clinical neuropathology service at Boston Children’s Hospital via standard protocols. Muscle samples were taken from Patient 1 (P1) at four years of age and from the quadriceps femoris of P2 at five years of age. The samples were then either frozen in isopentane cooled in liquid nitrogen or embedded in paraffin. Serial frozen sections (10-μm thick) were subjected to hematoxylin & eosin, nicotinamide adenine dinucleotide-tetrazolium reductase (NADH-TR), or myosin-ATPase staining.

### Protein Stability Assay of DNAJB4 Variants

As previously reported [5, 8], Flp-In T-REX 293 cells (Invitrogen) harboring pcDNA5/FRT/TO vectors encoding V5-tagged DNAJB4 constructs, including wild-type (WT), R183* K35N, K286*, and R61G variants, were utilized. These cells were maintained in Dulbecco’s modified Eagle’s medium (DMEM) supplemented with 4 mM L-glutamine (Invitrogen; 11965–084), 10% fetal bovine serum (FBS; Atlanta Biologicals; S10350), and a combination of 50 IU/ml penicillin and 50 μg/ml streptomycin (Invitrogen; 15140). The culture medium also included 50 μg/ml hygromycin B (Invitrogen; 10687–010) and 50 μg/ml blasticidin (Life Technologies; R21001) to ensure the selection of stably integrated constructs. The expression of the *DNAJB4* variants was induced with 1 μg/ml tetracycline hydrochloride (Sigma; T76600) 48 hours before the experiments were conducted. The cell cultures were incubated in a 5% CO2 atmosphere at 37°C until they reached 80–85% confluency in 60 mm tissue culture-treated dishes.

### TDP-43 disaggregation assay

The samples were processed as described in the literature [4, 5, 8, 9]. Briefly, HeLa cells were cultured in DMEM supplemented with 10% FBS and penicillin/streptomycin. Transfection was performed via Lipofectamine 2000 (Thermo Fisher Scientific) according to the manufacturer’s instructions. The cells were either transfected with mCherry-tagged TDP-43 alone or cotransfected with mCherry-tagged TDP-43 and GFP-tagged DNAJB4 constructs. The *DNAJB4* variants evaluated included GFP-DNAJB4-WT, GFP-DNAJB4-R183*, GFP-DNAJB4-K35N, GFP-DNAJB4-R259*, and GFP-DNAJB4-R61G, which were generated via the QuikChange Site-Directed Mutagenesis Kit (Agilent Technologies). Twenty-four hours after transfection, the cells were subjected to heat shock at 42°C for 1 hour, followed by a 2-hour recovery period at 37°C. The percentage of cells with TDP-43 nuclear aggregates was quantified after heat shock and recovery via fluorescence microscopy.

### Yeast Complementation Assay

In this study, the yeast strain utilized was derived from *Saccharomyces cerevisiae* 74-D694 (ade1–14 his3-Δ200 leu2–3 112 trp1–289 ura 3–52), with Sis1 gene deletion (sis1Δ:HygBMX4) performed as previously documented [5, 10]. Yeast cells were cultured either in rich YPD media (containing 1% yeast extract, 2% peptone, and 2% dextrose) or in synthetic defined (SD) media (composed of 0.67% yeast nitrogen base without amino acids and 2% dextrose), which lack certain nutrients to facilitate the selection of specific plasmids. Transformation of the cells was achieved via the plasmids pRS424-EV (empty vector), pRS424-HDJ1 (DNAJB1-WT), pRS424-HDJ1K35N (DNAJB1-K35N), pRS424-HDJ1R61G (DNAJB1-R61G), pRS414-Sis1WT, pRS414Sis1-K37N, and pRS414Sis1-R61G, employing the polyethylene-glycol/lithium-acetate (PEG/LioAC) technique, followed by selection on SD-trp agar plates. For the yeast spotting assay, cultures were incubated overnight in YPD media, collected via centrifugation, washed, and resuspended in sterile water to achieve an optical density of 0.1. These normalized cell suspensions were then aliquoted into a 96-well plate, and a series of 1:5 serial dilutions were prepared via a multichannel pipette. These dilutions were subsequently spotted onto both YPD agar plates and plates containing 1 mg/mL 5-fluoroorotic acid (5-FOA), the latter to select against cells harboring URA3-marked plasmids and to facilitate the replacement of the wild-type Sis1 with mutant constructs. This was accomplished via an ethanol-sterilized 48-pin replicator. The plates were then incubated at 30°C for five days before growth evaluation.

### Statistics

All the data are presented as the means ± SEMs, except for the age of onset, which is reported as the median. Pairwise comparisons for categorical variables between groups were made via Fisher’s exact test. For the age of onset, the Mann–Whitney U test was used to account for its numerical nature. We hypothesized that variants in the J-domain of *DNAJB4* are associated with a more severe phenotype than variants in other regions. A one-tailed test was used to evaluate this hypothesis. For the experimental data, multiple group comparisons were conducted via Student’s t test followed by Bonferroni correction. All the statistical analyses were executed via GraphPad Prism version 10 (GraphPad Software). A P value of less than 0.05 was considered statistically significant.

## Results

### Clinical features

**Figure 1:**
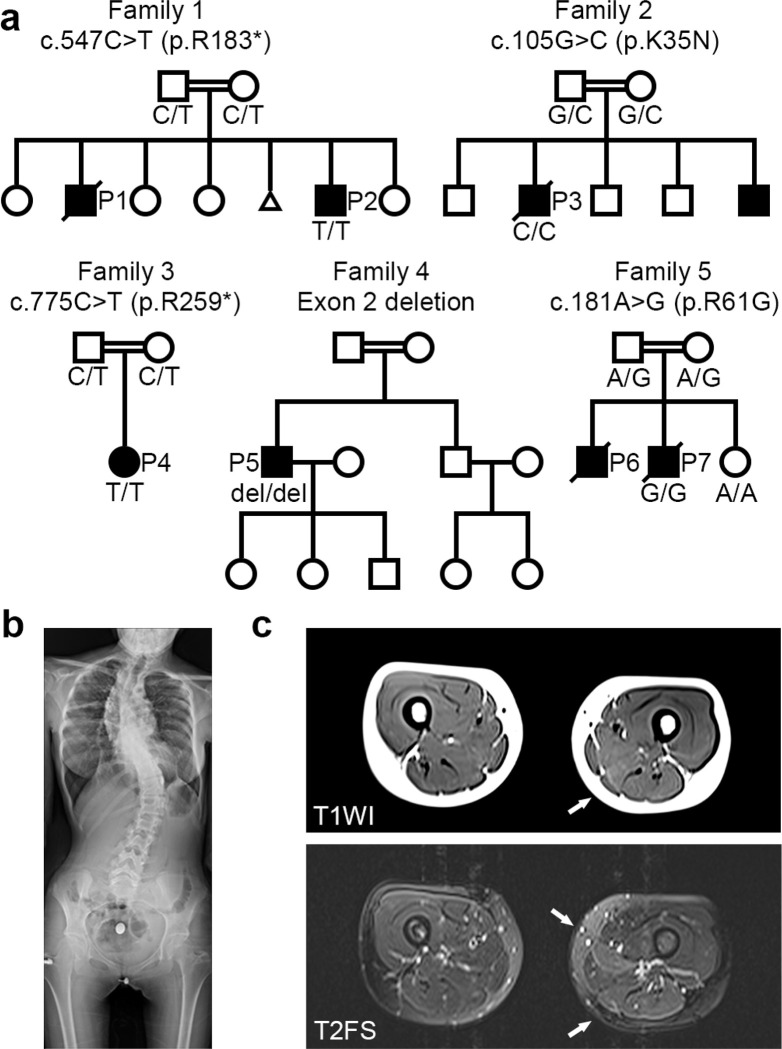
Pedigrees of patients with *DNAJB4* variants. (**a**) Pedigrees of five families demonstrating consanguinity and recessively inherited myopathy. The genotype of the patient(s) is represented with the pedigree. Below each symbol, the genotype is annotated: “del” indicates a deletion, and “ref” denotes the reference sequence. (**b**) X-ray image of P4 at 15 years of age, showing thoracic lumbar scoliosis. (**c**) Muscle magnetic resonance images of P3 at 14 years of age. Axial thigh muscle images of T1-weighted image (T1WI, upper image) and T2-weighted fat-saturated image (T2FS, lower image). High intensity in the semimembranosus muscle (arrows) is observed in both T1WI and T2FS, whereas the adductor longus shows high signal intensity only in T2FS (arrow).

Detailed clinical information for all patients, which included the five families in this study, is summarized in Table 1. Six of the patients were male, and one was female, with onset during the neonatal period and infancy. All the families were consanguineous (Fig. 1a). All the affected individuals experienced respiratory failure and required mechanical ventilation. Four patients had a history of recurring lower respiratory tract infections (P1, P4, P6, P7). Spinal rigidity was observed in three patients. Three patients had scoliosis, one of whom was born at birth (Fig. 1c). Three patients experienced neck stiffness from infancy. Ankle contractures were observed in three patients. Limb muscle weakness was not pronounced except in two patients: P1, who had predominantly distal weakness, and P5, who had predominantly proximal weakness. Three patients had dysphagia, all of whom required nasogastric or gastrostomy tubes. No apparent cognitive dysfunction was observed; however, on MR imaging, P3 had frontal lobe atrophy, and P7 had thinning of the corpus callosum and mild brain atrophy. No facial muscle involvement was observed.

Serum creatine kinase was within the normal range in all patients except for P4. Electromyography was performed on two patients (P4 and P5), and the results indicated myopathic changes, characterized by short-duration, low-amplitude, polyphasic motor unit potentials and early recruitment patterns. Cardiac dysfunction was observed in three patients—two with hypertrophic cardiomyopathy and one with mitral insufficiency.

### Spine X-ray and muscle imaging

The X-ray image of P4 from Family 3 at the age of 15 demonstrated thoracolumbar scoliosis (Fig. 1b). T2-weighted fat-saturated MR images of P4 at the age of 14 years showed high signal intensities in the semimembranosus and adductor longus muscles, whereas T1-weighted images revealed high signal intensity only in the semimembranosus muscle (Fig. 1c), suggesting earlier involvement of the semimembranosus muscle.

### Myopathological findings

**Figure 2:**
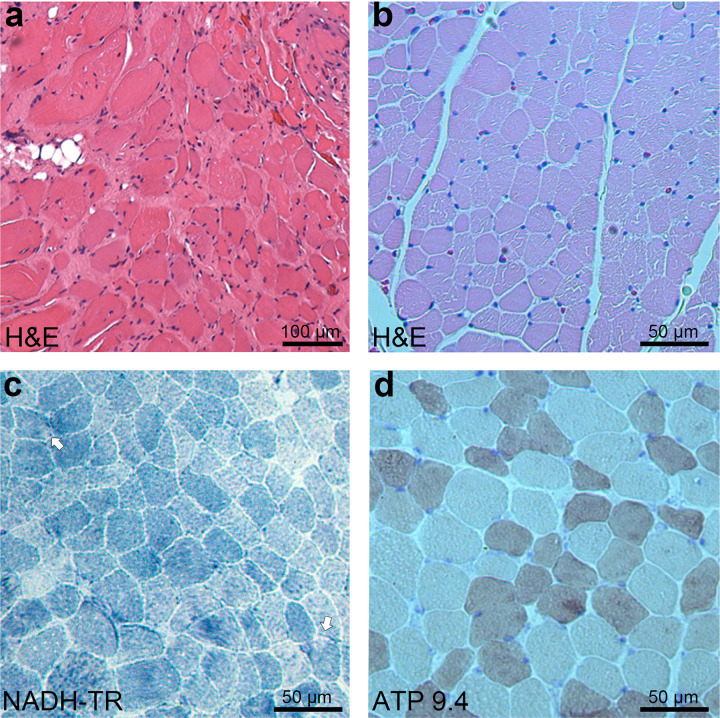
Myopathological findings from patients with *DNAJB4* variants. (**a**) H&E staining from P1 at 4 years of age, demonstrating marked variation in fiber size, prominent endomysial fibrosis, and adipose tissue infiltration. Scale bar: 100 μm. (**b**-**d**) Quadriceps biopsy from P2 at 5 years of age. (**b**) H&E staining showing moderate variation in fiber size and a few small angular fibers. Scale bar: 50 μm. (**c**) NADH-tetrazolium reductase (NADH-TR) staining highlighting small rubbed out areas (arrows). Scale bar: 50 μm. (**d**) ATPase staining at pH 9.4 demonstrating mild type 2 fiber atrophy. Scale bar: 50 μm.

Muscle biopsies were obtained from P1 at four years of age and P3 at five years of age. On H&E of the muscle from P1, notable endomysial fibrosis and adipose tissue infiltration were observed, with atrophic muscle fibers occasionally clustering (Fig. 2a). In the muscle of P3, in addition to moderate variation in fiber size (Fig. 2b), small regions with diminished enzyme activity were occasionally observed (Fig. 2c), and mild type 2 fiber atrophy was noted (Fig. 2d).

### Identification of DNAJB4 variants

**Figure 3:**
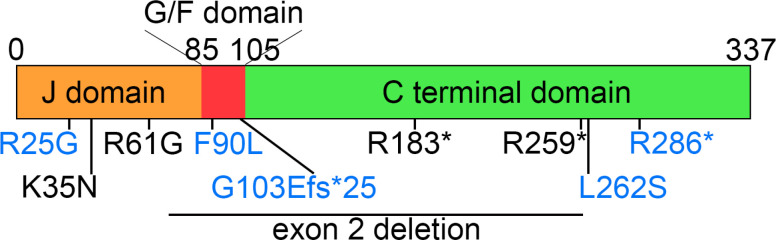
Schematic representation of DNAJB4 domains and variant locations. The schematic illustration of DNAJB4 shows the J-domain, glycine-phenylalanine rich (G/F) domain, and C-terminal domain. The locations of identified pathogenic variants are marked in black, whereas previously reported variants are indicated in blue.

We identified five distinct variants in *DNAJB4* (NM_007034.5) across Families 1 through 5: c.547C > T (p.R183*) in Family 1; c.105G > C (p.K35N) in Family 2; c.775C > T (p.R259*) in Family 3; an exon 2 deletion (hg38 chr1:78,013,051–78,013,619) in Family 4; and c.181A > G (p.R61G) in Family 5 (Fig. 3). All affected patients were homozygous for the indicated *DNAJB4* variant, and consanguinity was noted in all the families, with heterozygous variants found in the parents of Families 1, 2, 3, and 5 (Fig. 1). Notably, these variants were absent or exhibited extremely low allele frequencies in the gnomAD database (v4.0.0) and were not found in homozygous form, with allele frequencies of 0.000008893 for c.547C > T, 0.000003181 for c.105G > C, 0.000002813 for c.775C > T, and absent for c.181A > G. The *in silico* predictions for the missense variants indicated a disease-causing potential, with high CADD scores for K35N (31.0) and R61G (29.4).

### Genotype–phenotype correlation

We conducted a genotype–phenotype correlation analysis using data from five families in this study (Table 1), along with data from four previously reported families (Table 2, Fig. 3)[5, 6]. We hypothesized that mutations in the J-domain might be associated with a more severe phenotype. Therefore, we categorized the subjects into two groups: those with J-domain variants and those with other variants. The latter group includes variants thought to act as loss-of-function mutations, including one missense variant in the C-terminal domain (p.L262S)[5].

Compared with other variants, the J-domain variants were associated with an earlier age of onset (0.5 vs 28 years, P = 0.035) and a higher mortality rate (75% vs 14%, P = 0.046). However, there were no significant differences between the groups in terms of other clinical features: rigid spine (75% vs 43%, P = 0.39), neck stiffness (75% vs 29%, P = 0.20), scoliosis (25% vs 28.6%, P = 0.72), ankle contractures (50% vs 14%, P = 0.28), dysphagia (50% vs 14%, P = 0.28), cardiac findings (0% vs 57%, P = 0.07), brain imaging abnormalities (50% vs 0%, P = 0.11), or recurrent respiratory infections (40% vs 29%, P = 0.58).

### DNAJB4 Stability in Isogenic Cell Lines

**Figure 4:**
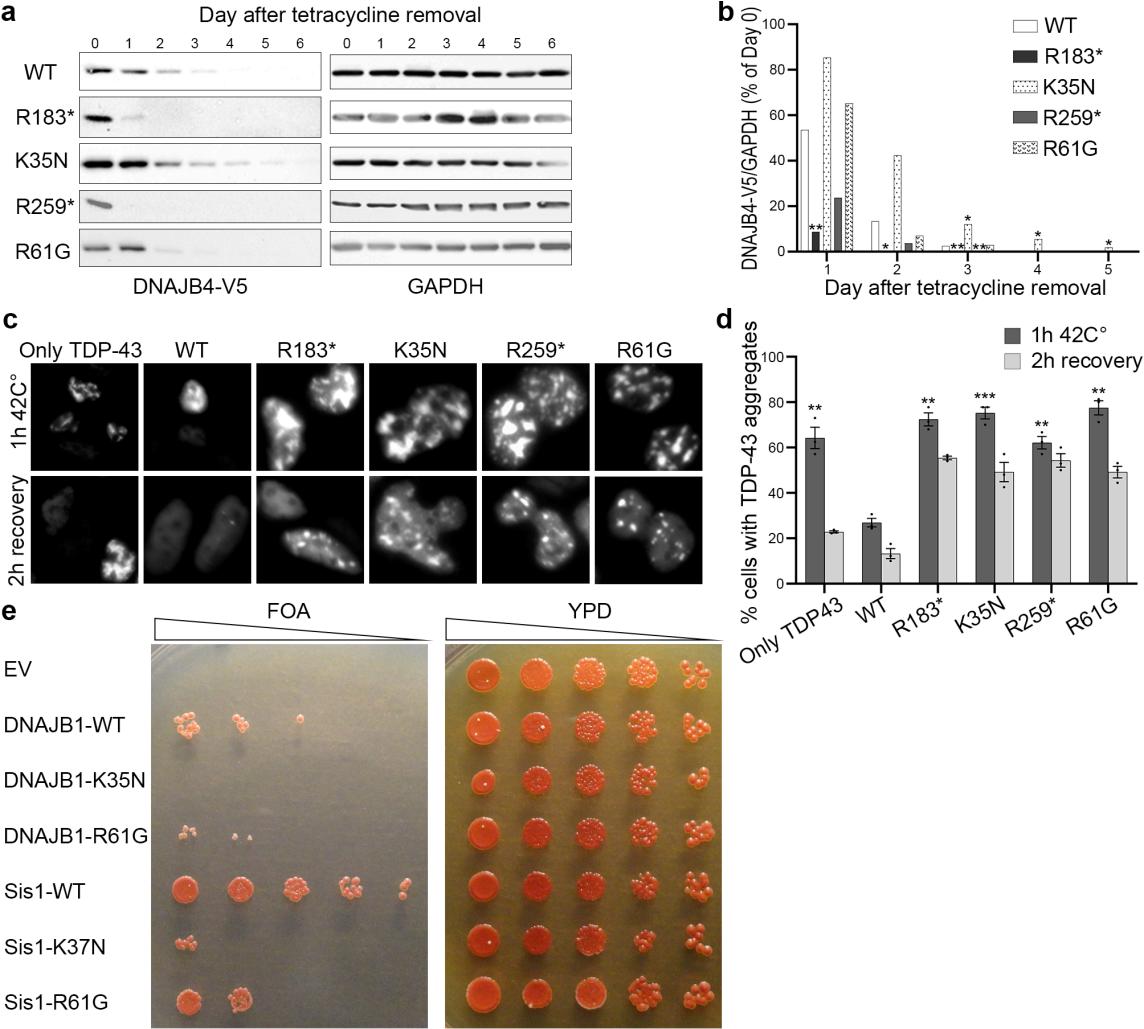
*DNAJB4* variants have a loss-of-function effect. (**a**) Tetracycline-regulated isogenic 293 cell lines were developed to express V5-tagged DNAJB4 (wild type, R183*, K35N, R259*, R61G). After induction with tetracycline for 24 hours, the cells were harvested at various times postinduction. Protein lysates were analyzed by immunoblotting for V5 and GAPDH, with GAPDH used as a loading control. (**b**) Densitometric analysis of V5-DNAJB4 normalized to GAPDH from three independent experiments, with the initial day’s value as a reference. Statistical significance was determined by comparing each variant to the WT on each day via a t test with Bonferroni correction for multiple comparisons (*P ≤ 0.0125, **P < 0.0025). (**c**) Representative fluorescence microscopy images displaying mCherry-tagged TDP-43 in HeLa cells post heat shock. (**d**) Quantification of cells displaying TDP-43 nuclear inclusions. *n = 200–300*cells analyzed per condition; the experiment was conducted 3 times. Statistical analysis was conducted by comparing the percentage of cells with TDP-43 aggregates after 1 hour at 42°C to that of WT cells via a t test with Bonferroni correction for multiple comparisons (**P < 0.002, ***P < 0.0002). (**e**) Yeast colonies lacking Sis1 were supplemented with either an empty vector (EV), wild-type DNAJB1 (DNAJB1-WT), DNAJB1-K35N, Sis1-WT, or the Sis1-K37N variant and then spotted on FOA media (left panel). The corresponding colonies on full media (YPD) are shown in the right panel. Data from five independent experiments were collected for each condition. EV, empty vector; WT, wild type.

Isogenic cell lines with stable integration of a tetracycline-regulatable V5-tagged DNAJB4 protein (V5-DNAJB4) were generated to express the reported variants [5]. Upon removal of tetracycline, wild-type DNAJB4 (DNAJB4-WT) was completely degraded within three days, as shown in Fig. 4a and b. As previously observed and expected, the truncation variants DNAJB4-R183* and DNAJB4-R259* were degraded more quickly, disappearing by the first and second days, respectively. Conversely, the DNAJB4-K35N variant exhibited enhanced stability compared with DNAJB4-WT, maintaining detectable levels beyond the degradation timeline of the wild-type protein. The DNAJB4-R61G variant did not significantly differ from DNAJB4-WT.

### TDP-43 disaggregation assay

To explore the impact of *DNAJB4* variants in a mammalian cell model, we evaluated their effects on TDP-43 aggregation. We previously reported that pathogenic variants in both *DNAJB4* and its homolog *DNAJB6*, which are associated with myopathy, alter the aggregation and disaggregation of their putative client protein, TDP-43 [4, 5, 8, 9]. HeLa cells were transfected either with mCherry-tagged TDP-43 alone or cotransfected with mCherry-tagged TDP-43 and GFP-tagged wild-type or mutant DNAJB4. We quantified the percentage of cells with TDP-43 nuclear aggregates after a 1-hour heat shock at 42°C and after a subsequent 2-hour recovery period.

Following the 1-hour heat shock, cells expressing myopathy-associated *DNAJB4* variants exhibited an increased percentage of cells with aggregates compared with those expressing DNAJB4-WT (Fig. 4c, d). Even after the 2-hour recovery period, a greater proportion of cells with aggregates was observed among the variant-expressing cells than among the wild-type-expressing cells.

### Yeast Complementation Assay

To investigate whether the DNAJB4-K35N variant and DNAJB4-R61G variant are dysfunctional, we utilized a yeast complementation assay as previously described [5, 8, 9].. Yeasts possess a homologous DNAJB protein, Sis1, which is crucial for viability. Both wild-type Sis1 (Sis1 WT) and human DNAJB1 wild-type (DNAJB1 WT) were able to compensate for the loss of yeast Sis1 (Fig. 4e). However, analogous mutants of K35N and R61G of DNAJB4 in Sis1 (Sis1-K37N and Sis1-R61G) and DNAJB1 (DNAJB1-K35N and DNAJB1-R61G) failed to rescue the knockout of Sis1. These findings suggest that the K35N and R61G variants in *DNAJB4* compromise its function.

## Discussion

In this study, we identified and characterized four novel pathogenic variants and one previously reported pathogenic variant in *DNAJB4* from five unrelated families of diverse ethnic backgrounds. The phenotypic manifestations are marked by severe respiratory complications and a broadened clinical spectrum that notably includes rigid spine syndrome. These findings expand our understanding of DNAJB4 myopathy, highlighting additional clinical features such as dysphagia, ankle contracture, scoliosis, neck stiffness, and cardiac dysfunction. Notably, our genotype–phenotype correlation analysis revealed that J-domain missense variants are associated with a more severe phenotype, including an earlier age of onset and a higher mortality rate. This genotype–phenotype correlation is supported by our functional study, which showed that null variants presented relatively low stability, whereas J-domain missense variants presented normal or increased stability but were dysfunctional in yeast complementation and TDP-43 disaggregation assays.

This study provides insight into the genotype–phenotype correlation in DNAJB4 myopathy. Patients with J-domain variants exhibit early onset and severe respiratory complications, often resulting in death before adulthood. These patients also presented a greater incidence of rigid spines, neck stiffness, ankle contractures, dysphagia, and recurrent respiratory infections. Notably, brain imaging abnormalities were observed exclusively in patients with J-domain variants. In contrast, patients with null variants, such as nonsense, frameshift, and exon deletions, exhibited later onset, with cardiac dysfunctions observed exclusively in these patients. These patients typically develop sudden respiratory failure without significant limb muscle weakness, usually in adulthood. Compared with null variants, functional analyses highlighted the distinct nature of J-domain missense variants. While null variants presented decreased protein stability, J-domain variants maintained normal stability but were dysfunctional in TDP-43 disaggregation and yeast viability assays. This suggests that J-domain variants may operate through a toxic gain-of-function mechanism, potentially via interactions with HSP70, similar to the dominant p.F90L variant [4]. However, since heterozygous carriers of these missense variants do not exhibit clinical symptoms, this remains speculative, underlining the complexity of the genotype–phenotype correlation in DNAJB4 myopathy.

The clinical features observed in our cohort significantly extend the known spectrum of recessive DNAJB4 myopathy. New symptoms of dysphagia, ankle contractures, and scoliosis were identified in three patients from three different families. Additionally, neck stiffness was identified from infancy in three patients, whereas cardiac dysfunction was observed in three patients. These findings are especially significant, as such symptoms have been scarcely documented in the context of DNAJB4 myopathy owing to the limited number of cases described. The detection of these clinical features in several new cases in the present study not only reveals that dysphagia, ankle contracture, and scoliosis are manifestations of DNAJB4 myopathy but also reinforces the understanding that neck stiffness and cardiac dysfunction are indeed manifestations of DNAJB4 myopathy. Given the pathogenic variants found in three families through reanalysis of whole-exome sequencing data, it is crucial to screen and reanalyze exome data for patients exhibiting these symptoms.

Acute respiratory failure with life-threatening hypercapnia necessitating prolonged ventilatory support is a hallmark of DNAJB4 myopathy and was a common symptom in all patients in this study and others [5, 6]. Our cohort indicates a polarization in the onset of DNAJB4 myopathy into two types: one presenting in infancy with early-onset neck stiffness, rigid spine syndrome with axial weakness out of proportion to limb weakness, and severe respiratory failure, and the other manifesting in adulthood with less apparent limb muscle weakness and lacking the characteristic complications seen in the pediatric type. The early-onset form, with its mild limb muscle weakness and lack of facial muscle involvement, should be differentiated from congenital myopathies, especially nemaline myopathy, neurogenic disorders such as spinal muscular atrophy, infantile-onset Pompe disease, congenital myotonic dystrophy, and congenital myasthenic syndrome, all of which can present in the neonatal period [11]. Differentiation from other early-onset conditions involving rigid spine syndrome caused by *SELENON, FHL1, LMNA*, and others is crucial [12–17]. Moreover, in adults, where muscle weakness is not pronounced, the differential diagnosis includes conditions such as hereditary myopathy with early respiratory failure (HMERF) due to *TTN* variants and myofibrillar myopathies [11, 18].

The present study enhances our understanding of the muscle pathology and skeletal muscle imaging of DNAJB4 myopathy. Both recessive and dominant forms of DNAJB4 myopathy are known to feature cytoplasmic inclusions [4, 5];; however, such inclusions were not observed in this study. The areas of diminished oxidative enzyme activity noted may suggest the presence of inclusions, but confirmation by immunostaining was not possible owing to limited access to muscle samples. In fact, the absence of inclusions in recessive DNAJB4 myopathy has been previously documented [5]. The type 2 fiber atrophy observed in this study is also consistent with the muscle pathology observed in a previous report [5] and may be one of the characteristic features of recessive DNAJB4 myopathy. Muscle MR images showing the involvement of the semimembranosus and adductor longus muscles extend the pattern of selective muscle involvement in this disease.

While muscle biopsies were not available for all patients, limiting direct pathological examinations, we successfully utilized cell-based models to assess myopathy-associated *DNAJB4* variants. This approach provides valuable insights into potential disease mechanisms, illustrating the difference between null variants and missense variants in *DNAJB4*, specifically with respect to protein stability. Notably, dominant variants in *DNAJB4* and *DNAJB6* have increased stability and unproductive interactions with HSP70, leading to a potentially gain-of-function mechanism [4, 8].

## Conclusions

DNAJB4 is an emerging cause of inherited myopathy with respiratory failure and rigid spine syndrome of variable age of onset and severity. It should be suspected in individuals with suggestive symptoms, especially if they present with neck stiffness during infancy or when respiratory failure is noted in adults without prominent limb muscle weakness. The discovery of new familial cases from diverse populations has contributed to expanding the clinical spectrum. It is prudent to screen patients with DNAJB4 myopathy regularly for early cardiac and respiratory manifestations and dysphagia and proactively institute life-saving supportive measures. J-domain missense variants may be associated with a severe clinical course. Understanding the relationships between diverse variants and phenotypes and elucidating their mechanisms will be pivotal in developing treatments for this devastating disease.

## Figures and Tables

**Table 1:** Clinical features of identified patients with *DNAJB4* variants.

	Family 1		Family 2	Family 3	Family 4	Family 5 [7]	
**Individual**	P1	P2	P3	P4	P5	P6	P7
**DNAJB4 variant**	Not tested	Hom c.547C > T (p.R183*)	Hom c.105G > C (p.K35N)	Hom c.775C > T (p.R259*)	Hom exon2 deletion^#^	Not tested	Hom c.181A > G (p.R61G)
**Ethnicity/Consanguinity**	Saudi Arabian/Yes		Saudi Arabian/Yes	Turkish/Yes	Colombian/Yes	Arab/Yes	
**Sex**	Male	Male	Male	Female	Male	Male	Male
**Age at last examination**	9 (deceased)	2	7 (deceased)	16	48	11 months (deceased)	7 (deceased)
**First symptoms (age)**	Neck stiffness (9 months)	Neck stiffness (5 months)	Neck stiffness (6 months)	Recurrent respiratory infection (1)	Muscle weakness (31)	Respiratory failure (3 months)	Respiratory failure (4 months)
**Respiratory failure**	Yes	Yes	Yes	Yes	Yes	Yes	Yes
**Rigid spine**	Yes	No	Yes	Yes	No	NA	No
**Neck stiffness**	Yes	Yes	Yes	No	No	NA	No
**Scoliosis**	Yes	No	Yes	Yes	No	NA	No
**Joint contractures**	Yes (ankle)	No	Yes (ankle)	No	No	NA	Yes (ankle)
**Dysphagia**	Yes	No	Yes	No	No	NA	Yes
**Cardiac finding**	HCM	No	No	MI	HCM	NA	No
**CK**	Normal	165	normal	1226	222	NA	Normal
**EMG**	Normal	NA	NA	Myopathic (Int. Poly, Short)	Myopathic (Poly. Short, Early Rec)	NA	NA

# hg38 chr1:78,013,051–78,013,619; P1, BOS_0868-1; P2, BOS_0868-4; P3, BOS_1575-1; NA = not available; Hom = homozygous; HCM = hypertrophic cardiomyopathy; MI = mitral insufficiency; CK = creatine kinase; EMG = electromyography; Int = interference; Poly = polyphasic pattern of motor unit action potential; Short = short duration of motor unit action potential; Early Rec = early recruitment.

**Table 2:** Clinical features of reported patients with *DNAJB4* variants.

	Family A [5]	Family B [5]	Family C [5]		Family [4]	Patient #60 [6]
**Individual**	AII:1	BII:1	CII:1	CII:2		
**DNAJB4 variant**	Hom c.856A > T (p.K286*)	Hom c.785 T > C (p.L262S)	Hom c.74G > A (p.R25Q)	Hom c.74G > A (p.R25Q)	Het c.270T > A (p.F90L)	Hom c.308del (p.G103Efs*25)
**Ethnicity/Consanguinity**	Spanish/no	Spanish/no	Saudi Arabian/yes		Japanese/no	NA
**Sex**	Male	Female	Female	Male	4 males, 2 females	Female
**Age at last examination**	33	63	11 (deceased)	6	49–75	39~
**First symptoms (age)**	Respiratory failure (28 years)	Respiratory failure (45 years)	Neck stiffness, scoliosis (1 year)	Neck stiffness, scoliosis (1 year)	Weakness of thumb (late 20 s – 30 s)	Weight loss (32 years)
**Respiratory failure**	Yes	Yes	Yes	Yes	Yes (60 s – 70 s)	Yes
**Rigid spine**	Yes	No	Yes	Yes	No	No
**Neck stiffness**	No	No	Yes	Yes	No	No
**Scoliosis**	No	No	No	No	No	No
**Joint contractures**	No	No	No	No	Yes (Fingers)	No
**Dysphagia**	No	No	No	No	No	No
**Cardiac finding**	No	HCM	No	No	No	No
**CK**	500–700	Normal	350–400	350–400	646–1285	Normal
**EMG**	NA	Myopathic	Myopathic	Myopathic	Myopathic	NA

NA = not available; Hom = homozygous; Het = heterozygous; HCM = hypertrophic cardiomyopathy; CK = creatine kinase; EMG = electromyography.

## Data Availability

The datasets generated and analyzed during the current study are available from the corresponding author on reasonable request.
